# Utilizing Undissolved Portion (UNP) of Cement Kiln Dust as a Versatile Multicomponent Catalyst for Bioethylene Production from Bioethanol: An Innovative Approach to Address the Energy Crisis

**DOI:** 10.1021/acsomega.3c09043

**Published:** 2023-12-28

**Authors:** Mahmoud Nasr, Adel Abdelkader, Safaa El-Nahas, Ahmed I. Osman, Amal Abdelhaleem, Hossam AbdelFattah El Nazer, David W. Rooney, Samih A. Halawy

**Affiliations:** † Nanocomposite Catalysts Laboratory, Chemistry Department, Faculty of Science at Qena, South Valley University, Qena 83523, Egypt; ‡ School of Chemistry and Chemical Engineering, Queen’s University Belfast, Belfast BT9 5AG, Northern Ireland, U.K.; § Environmental Engineering Department, Egypt-Japan University of Science and Technology (E-JUST), Alexandria 21934, Egypt; ∥ Photochemistry Department, National Research Center, Dokki, Giza 12622, Egypt

## Abstract

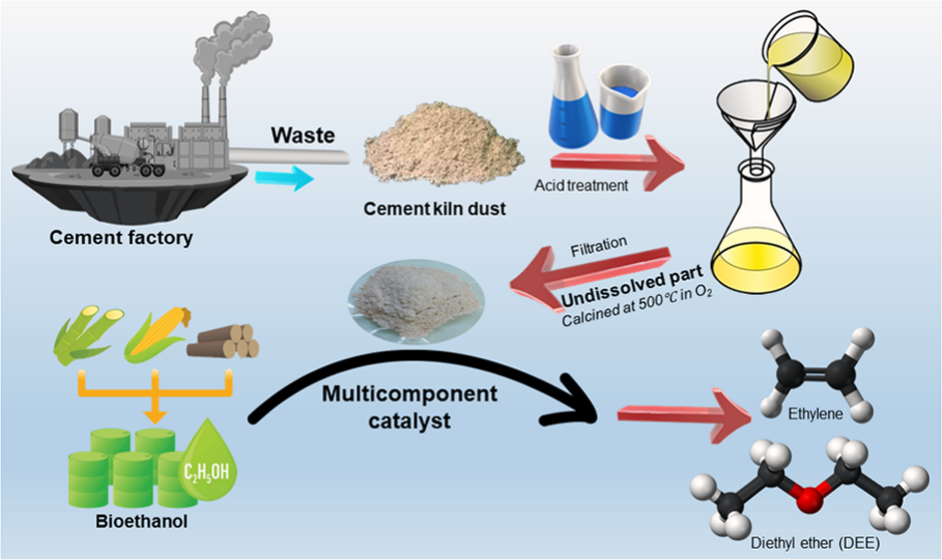

This study focuses on upcycling cement kiln dust (CKD) as an industrial waste by utilizing the undissolved portion (UNP) as a multicomponent catalyst for bioethylene production from bioethanol, offering an environmentally sustainable solution. To maximize UNP utilization, CKD was dissolved in nitric acid, followed by calcination at 500 °C for 3 h in an oxygen atmosphere. Various characterization techniques confirmed that UNP comprises five different compounds with nanocrystalline particles exhibiting an average crystal size of 47.53 nm. The UNP catalyst exhibited a promising bioethylene yield (77.1%) and selectivity (92%) at 400 °C, showcasing its effectiveness in converting bioethanol to bioethylene with outstanding properties. This exceptional performance can be attributed to its distinctive structural characteristics, including a high surface area and multiple-strength acidic sites that facilitate the reaction mechanism. Moreover, the UNP catalyst displayed remarkable stability and durability, positioning it as a strong candidate for industrial applications in bioethylene production. This research underscores the importance of waste reduction in the cement industry and offers a sustainable path toward a greener future.

## 1 Introduction

The cement industry plays a vital role in the economic development of a country. It experienced a rapid growth phase in the 20th century, and its production capacity reached its peak and remained stable in this century.^1^ Global cement production has consistently and significantly increased due to population growth, ongoing urbanization, and infrastructure development. By 2050, the total annual cement consumption is projected to increase from 4.13 Gt in 2016 to 4.68 Gt.^2^ Although cement production is a crucial industry for sustainable growth in many countries, it results in significant amounts of toxic waste every day,^3^ including cement kiln dust (CKD) and CO_2_ emissions.^4^ CKD, which is a byproduct produced when clinker is manufactured, contains notable quantities of alkali, sulfate, and chlorides. A considerable portion of CKD, which has lower levels of alkali, sulfate, and chloride, is repurposed as a raw material in cement kilns. However, remaining CKD is currently deposited in landfills. Although advancements in the cement manufacturing process have led to significant decreases in CKD production, there is still a substantial amount of CKD that ends up in landfills.^5^ Approximately 80% of CKD production is stockpiled or landfilled, causing significant environmental issues.^6^ Toxic waste such as CKD can harm people, animals, and plants, whether it ends up in the ground, in streams, or even in the air. Dangerous effects of CKD come from its strong alkalinity and its high content of heavy metals.^7^ Disposing of CKD results in the utilization of land and also leads to the pollution of both surface and groundwater due to the release of chemicals and heavy metals that leach out from the CKD.^8^

Furthermore, CKD has a negative impact on human health by producing major health problems such as asthma, skin irritation, and eye problems.^9^ Therefore, the Earth is facing a severe environmental disaster due to global warming caused by the steady rise of atmospheric CO_2_ emissions.^10^ In May 2022, the Scripps Institute of Oceanography at the University of California released data indicating that the daily average concentration of atmospheric CO_2_ reached an unprecedented height of 421.37 ppm.^11^ Presently, the cement industry ranks as the third largest consumer of industrial energy, and its production is responsible for approximately 6–7% of global anthropogenic CO_2_ emissions.^2^ The production of one ton of cement clinker results in the generation of approximately 54–200 kg of CKD, along with the release of 600–700 kg of CO_2_ gas into the environment.^4,12^ These data suggest that the situation is becoming more concerning and urgent action is required to address the issue. Currently, solid waste management practices are considered a crucial issue confronting all countries throughout the world.^13^ In this context, recycling and waste management of CKD play crucial roles in minimizing environmental impact and maximizing resource efficiency.

The global economy has been notably affected by both the COVID-19 pandemic and the Russia–Ukraine conflict, with a pronounced impact on the energy sector. The pandemic brought about sudden shifts in energy demand, causing shocks in oil prices, disruptions in supply chains, and decreases in investment. Conversely, the conflict has led to elevated energy prices and posed challenges to energy security.^14,15^ While the pandemic primarily affected energy demand on a systemic scale, the conflict directly influenced energy production, supply, and trade. Actions taken by individuals and nations to mitigate supply disruptions, impose sanctions, and reduce import dependency also impact energy demand.^14,16^ Energy sources are considered essential to modern industries and play a crucial role in a country’s economy. However, the finite reserves of fossil fuels and growing environmental pollution have become significant obstacles to further economic development.

Consequently, finding safe and environmentally friendly energy sources has become a growing concern in establishing sustainable development.^17^ Moreover, renewable energy sources have a lower impact on the environment compared with conventional sources, reducing greenhouse gas emissions and mitigating climate change. This shift toward renewable energy also creates new job opportunities and stimulates economic growth in the renewable energy sector.^18^

Biofuels derived from renewable sources, in particular, are regarded as one of the most promising energy sources for the transportation sector.^17^ Due to their environmental concerns, energy safety, and socioeconomic index, biofuels are deemed a significant renewable energy source worldwide. Gaseous or liquid fuels that are primarily derived from biomass are generally referred to as “biofuels”.^19^ In particular, bioethanol, produced from various substrates,^20^ is becoming more widely available as a feedstock, and thus it was utilized in various processes, such as hydrogen, ethylene, and diethyl ether (DEE) production. Ethylene production is a mature technology in the energy sector, and the demand for renewable ethylene is steadily increasing due to economic growth, with the ethylene market growing by approximately 4% annually. Given the increasing availability of ethanol from renewable sources, alternative routes to ethylene production have been developed. Bioethanol-to-bioethylene processes have been designed based on this availability, and they are becoming economically sustainable, especially in light of the recent increase in oil prices.^21,22^

Ethylene is referred to as the “king of petrochemicals” due to its several advantageous qualities, as well as other technological and financial factors, as more commercial chemicals are created from ethylene than from any other intermediate.^23,24^ It is used as a raw material to make ethylene glycol as well as solvents, surfactants, plasticizers, detergents, and plastics. From the ethylene platform molecule, a variety of high-volume plastics can be produced, different grades, including polystyrene (PS), polyvinyl chloride (PVC), polypropylene (PP), polyethylene terephthalate (PET), and polyethylene (PE).^25^ Additionally, acetaldehyde, acetic acid, ethylene glycol, ethylbenzene, styrene, and vinyl acetate are all manufactured using ethylene as a raw material.^26^ The global annual ethylene (C_2_H_4_) production reached 185 Mt in 2018.^27^ The main ethylene production regions are North America, Western Europe, the Middle East, and North East Asia.^28^ At present, the conversion of bioethanol into ethylene shows great potential because of the increasing cost of ethylene and the renewable characteristics of bioethanol.^29^ The method of ethylene production varies depending on the feedstock, and it involves either steam cracking ethane or naphtha. These processes are known for their substantial energy usage and carbon emissions, resulting in the generation of 1/2 tonne of CO_2_ for each tonne of ethylene produced. This is primarily due to the significant energy demand of cracking reactions and the intricate cryogenic separation procedures involved.^30^ As a result of its sustainability, bioethanol dehydration has been promoted as an attractive way to produce ethylene.^24,31,32^ Compared to using fossil feedstock, the process of producing ethylene through the dehydration of ethanol results in an environmental benefit of 32.0 MJ of energy and the prevention of 1.87 kg of CO_2_ from being released into the atmosphere.^32^ On the other hand, diethyl ether (DEE) is considered a promising alternative fuel or an oxygen supplement for diesel engines, owing to its advantageous properties, such as a high cetane number and oxygen content. Furthermore, DEE’s ability to remain in a liquid state at typical environmental temperatures renders it an attractive option for the storage and transportation of fuel.^33^

In view of the above, this study could provide a real and vital contribution to alleviating the two crucial problems confronting Egyptian society and all countries throughout the world: the solid waste problem and the energy problem. The key advancements presented in this study can be encapsulated in the following key points: (1) the recycling of CKD waste, thereby mitigating the cement industry’s negative environmental impacts and (2) the utilization of the undissolved part (UNP) extracted from CKD as a catalyst in the synthesis of bioethylene from bioethanol after a suitable thermal treatment as a unique solution for the energy crisis.

## 2 Experimental Section

### 2.1 Materials

In this study, we utilized a specimen of CKD obtained from the Misr Qena Cement Plant in Qeft, Qena governorate. The chemical composition of the CKD specimen was determined by the Cement Plant, and the results are provided in Table S1. Nitric acid (HNO_3_, 50–55%), oxalic acid (H_2_C_2_O_4_·2H_2_O), and ammonia solution (25%) of analytical grade purity were purchased from El Nasr Pharmaceutical Chemicals Co. Egypt - ADWIC.

### 2.2 Estimation of the UNP and the Amount of CaO in the CKD Sample

To estimate the amount of CKD that did not dissolve in nitric acid, 5 g of CKD sample was mixed with 50 mL of deionized water, and the mixture was continuously stirred using a magnetic stirrer at room temperature. Then, 12.5 mL of nitric acid (50–55%) was added dropwise to the mixture until the effervescence stopped. The mixture was filtered through Crucible Gooch Sintered Glass-G3 to separate the undissolved part. The UNP was dried at 120 °C for 12 h, and its weight was about 8.6% of the original CKD sample. The UNP obtained was calcined at 500 °C for 3 h in an oxygen flow of 100 mL·min^–1^. To determine the amount of CaO in CKD, 100 mL of oxalic acid (4%) was added to the filtrate obtained in the previous step, and then dilute aqueous ammonia solution was added until the pH reached 8.5–9. A precipitate of calcium oxalate (CaC_2_O_4_·H_2_O) was formed, which was boiled and immediately filtered through ashless filter paper. Finally, the precipitate was dried and subjected to calcination at 500 °C for 2 h in a muffle furnace to produce calcium carbonate (CaCO_3_). The weight of the produced CaCO_3_ was equivalent to 2.5 g of CaO, which is about 54.7% of the dissolved portion and 50% of the original CKD sample.

### 2.3 Characterization Techniques

The UNP was determined by XRD, FT-IR, EDXRF, SEM, XPS, HR-TEM, elemental mapping, and surface area measurement (*S*_BET_) analyses. To quantify the overall quantity of acidic sites (sites·g^–1^) on the UNP sample that had undergone calcination at 500 °C, a temperature-programmed desorption (TPD) technique was applied to the condensed phase of tetrahydrofuran (THF). The measurement of the mass loss resulting from THF desorption during thermogravimetric (TG) experiments from the acidic sites was employed to measure the total surface acidity, quantified as sites·g^–1^. The estimation of the total count of surface acidic sites was achieved using eq 1:^4,34^
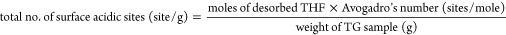
1For more details about characterization techniques, see the Supporting Information.

### 2.4 Catalytic Activity of UNP Calcined at 500 °C during the Decomposition of Bioethanol

The catalytic activity of the synthesized catalyst for the vapor phase dehydration of bioethanol obtained from the El-Hawamdia factory for the integrated sugar industry was performed in a Pyrex glass reactor (1 cm wide and 16.5 cm long) under 50 mL·min^–1^ flow of a mixture of nitrogen and air in equal proportions, i.e., (25 mL·min^–1^ N_2_ + 25 mL·min^–1^ air) as a carrier gas. The catalytic activity and selectivity of catalyst samples for decomposing bioethanol to products, mostly in the temperature range of 300–400 °C, were investigated. 0.5 g of catalyst sample was preheated at 450 °C inside a fixed-bed continuous flow reactor for 1 h under the air-nitrogen stream before measurements; then, the temperature was gradually decreased to 300 °C. Bioethanol vapors were generated by passing the air-nitrogen stream through the liquid bioethanol in a glass saturator thermostatically stabilized at 0 °C. The gas hourly space velocity of 6 L·g_cat_^–1^·h^–1^ was used in all of the experiments. The anticipated reaction products, such as ethylene, DEE, and acetaldehyde, were analyzed and detected using an online gas chromatograph (Shimadzu GC-14) equipped with a data processor model Shimadzu Chromatopac C-R4AD. Continuous automatic sampling was carried out using a heated gas sampling cock (type HGS-2) at 140 °C. A hydrogen flame ionization detector (FID) and a stainless-steel column (PEG20 M, 20% on Chromosorb W, 60/80 mesh, 3 m × 3 mm) maintained at 75 °C were employed. The conversion percentage of bioethanol and the selectivity percentage of the products were calculated using eqs 2 and 3:^35^
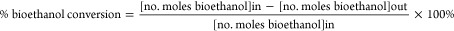
2
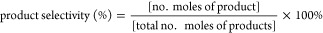
3[No. moles bioethanol]in and [No. moles bioethanol]out denote the number of bioethanol moles in the feed and outlet streams, respectively.

## 3 Results and Discussion

### 3.1 Characterization

#### 3.1.1 Thermal Analyses

**Figure 1:**
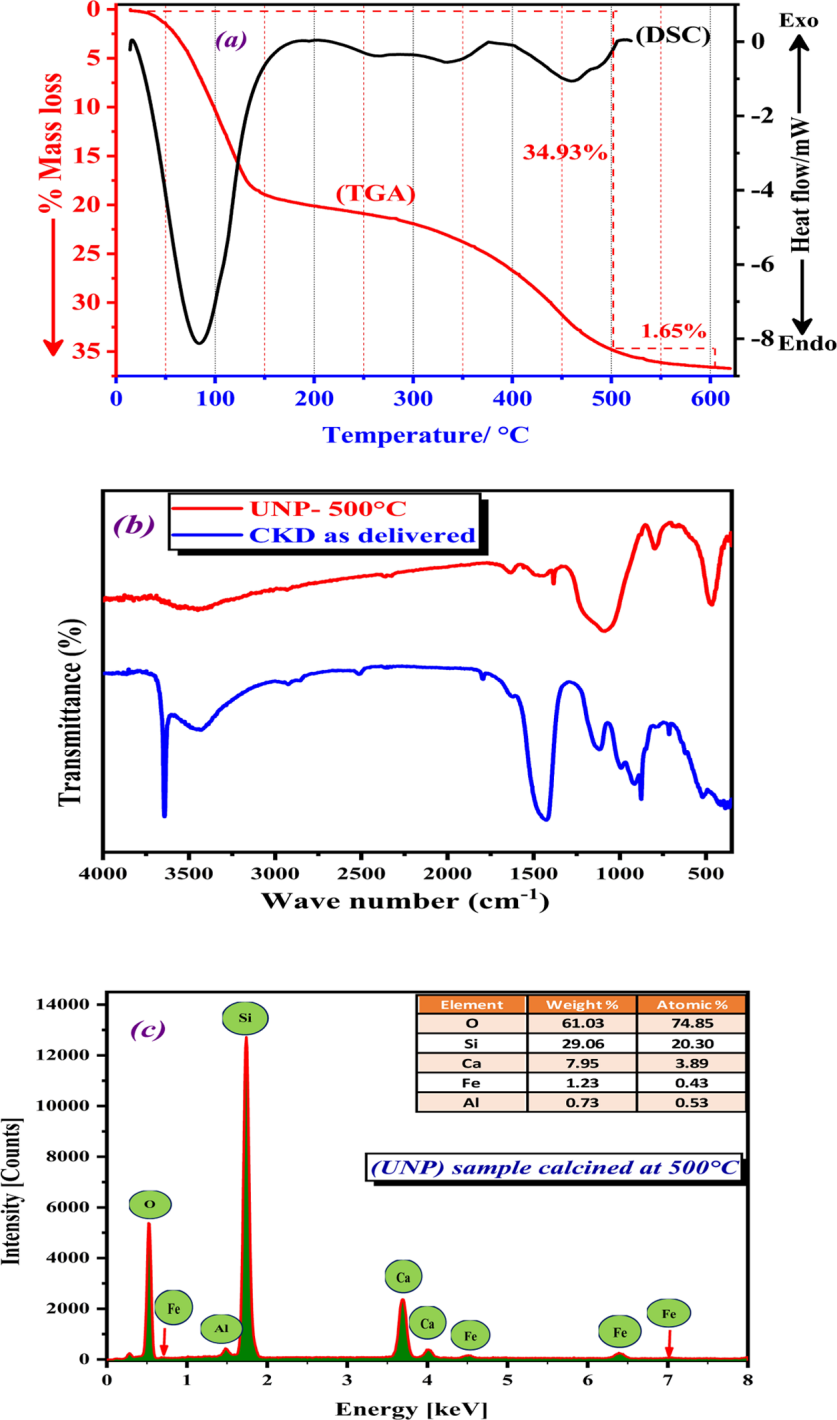
(a) TG and DSC profiles of undissolved UNP sample prepared from CKD were performed in 40 mL·min^–1^ N_2_, with 10 °C min^–1^ heating rate, (b) FT-IR spectra of CKD as delivered and UNP sample prepared from CKD calcined at 500 °C for 3 h in an oxygen flow of 100 mL·min^–1^, and (c) EDX spectrum of UNP sample calcined at 500 °C prepared from CKD for 3 h in an oxygen flow of 100 mL·min^–1^.

Thermogravimetric analysis (TGA) and differential scanning calorimetry (DSC) techniques are used to investigate the thermal stability of the UNP catalyst prepared from CKD during thermal treatment. The thermal analysis is carried out in a dry N_2_ atmosphere (40 mL·min^–1^) with a heating rate of 10 °C·min^–1^. As displayed in Figure 1a, the TGA curve of the UNP sample shows two mass loss steps from room temperature (RT) to 600 °C with a total mass loss of 36.58%. In the RT-500 °C temperature range, the consecutive steps in the TGA profile show a mass loss of 34.93%. Furthermore, a very tiny weight loss step of 1.65% is observed between 500 and 600 °C. The first mass loss (21%) step in the TGA curve between RT and 250 °C can be attributed to the removal of the surface adsorbed water. The second mass loss step (15.58%), which is observed in the 250–600 °C temperature range, could be related to the elimination of residual nitrates.^4^ The DSC curve of the UNP sample exhibited four endothermic peaks. The first step in the DSC curve (main peak) was located at 85 °C, related to the removal of the adsorbed water on the surface of the UNP sample. The second, third, and fourth endothermic peaks (minor broad peaks) in the DSC curve were located at 260, 336, and 461 °C are related to the decomposition of the residual nitrates. According to the thermal analysis results, the calcination at 500 °C for 3 h in oxygen flow is suitable to make a thermally stable UNP sample.

#### 3.1.2 X-ray Powder Diffraction (XRD) Analysis

**Figure 2:**
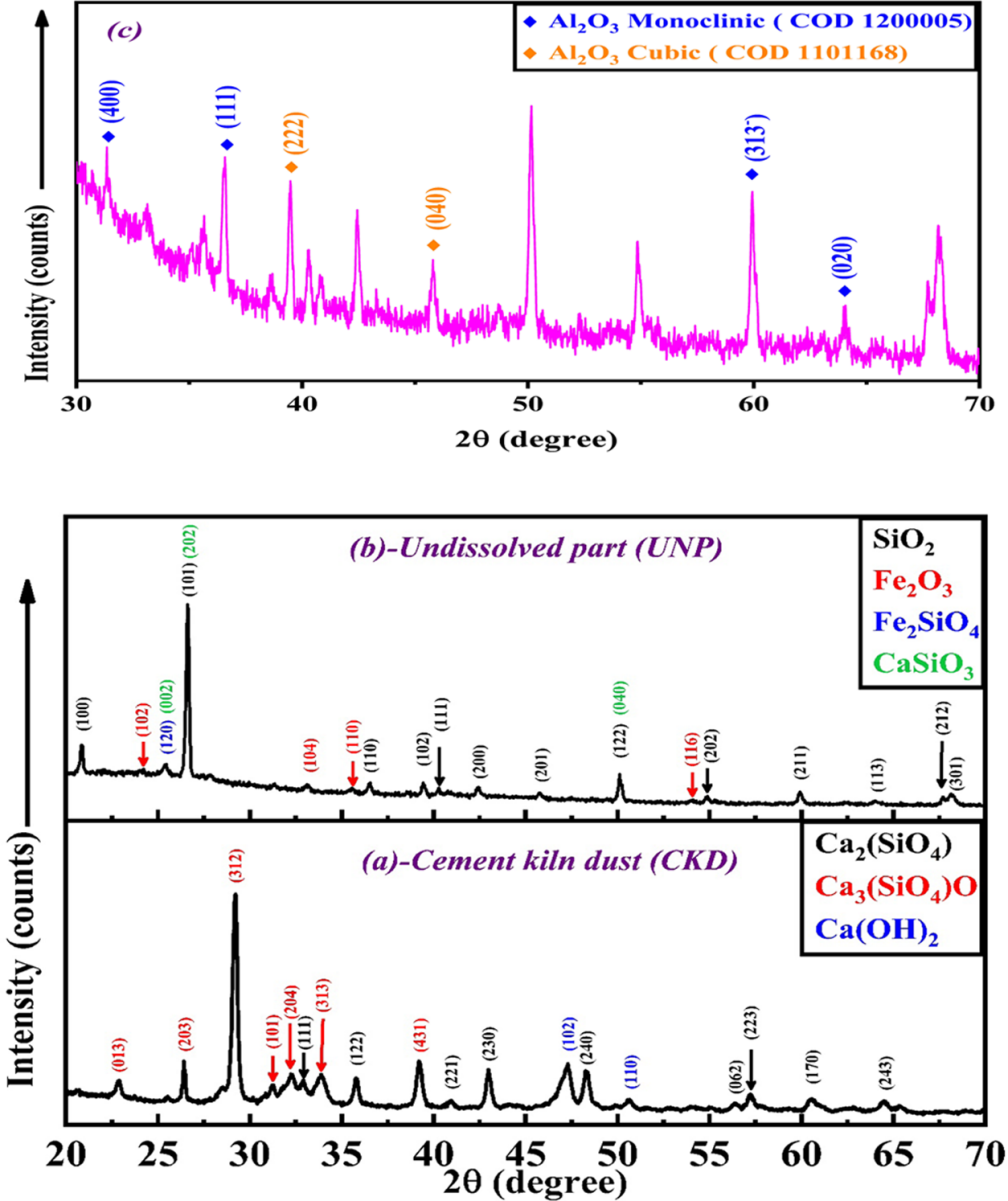
XRD examination of the original CKD and the UNP sample from the CKD for comparison. (a) XRD diffraction patterns of CKD as delivered, (b) XRD diffraction patterns of UNP sample from CKD calcined at 500 °C for 3 h in an oxygen flow of 100 mL·min^–1^, and (c) XRD diffraction patterns of UNP sample calcined at 500 °C from CKD for 3 h in an oxygen flow of 100 mL·min^–1^ in the range of 2θ = 30–70°.

One of the most effective methods for structural analysis of materials at the micro- and even nanoscales is the X-ray diffraction (XRD) technique. To dissect the phase structure and crystallite size of this sample, XRD analysis has been performed. Figure 2 shows an XRD examination of the original CKD and the UNP sample from CKD for comparison. The XRD diffractogram of CKD as delivered, as discussed in our recently published research,^4^ shows the three main constituents of CKD: tricalcium silicate or hartrurite (C_3_S) [Ca_3_(SiO_4_)O], dicalcium silicate or lamite (C_2_S) [Ca_2_SiO_4_], and calcium hydroxide or portlandite [Ca(OH)_2_], see Figure 2a. As shown in Figure 2b,c, the XRD diffractogram of the UNP sample calcined at 500 °C revealed the presence of five compounds. These compounds include (SiO_2_), (CaSiO_3_), (Fe_2_O_3_), (Fe_2_SiO_4_), and (Al_2_O_3_). The primary component of the UNP sample was identified as silicon dioxide (SiO_2_) (COD 7103014) via the intense miller index (101) at 2θ = 26.65°, as well as through the observation of 12 other planes at (100), (110), (102), (111), (200), (201), (112), (202), (211), (113), (212), and (203), corresponding to 2θ of 20.86, 36.56, 39.47, 40.3, 42.4, 45.8, 50.1, 54.8, 59.9, 64.1, 67.7, and 68.1°, respectively.^36^ These peaks, depicted in Figure 2b, appear sharp, indicating the high crystallinity of the silicon dioxide. Additionally, four diffraction peaks related to iron (III) oxide (COD 2101167) were detected at 2θ = 24.1, 33.1, 35.6, and 54.09°, which correspond to (102), (104), (110), and (116) planes, respectively.^37^ Three other diffraction lines (002), (202), and (040) belonging to calcium silicate (CaSiO_3_) appeared at 2θ = 25.2, 26.7, and 50.1° (COD 9011913).^38^ The presence of calcium silicate confirms that the extraction of calcium from the cement kiln dust is sufficient because the remainder is in the form of calcium silicate, and this confirms that the CKD sample is a rich source of calcium. Furthermore, a small diffraction peak correlated to fayalite (Fe_2_SiO_4_) appeared at 2θ = 25.4°, which corresponds to the (120) plane.^39^ To get a deep look and examine the diffraction peaks related to alumina (Al_2_O_3_) in better detail in the XRD diffractogram, the XRD diffractogram of (UNP) sample calcined at 500 °C in the range of 2θ = 30–70° is presented in Figure 2c. Six diffraction peaks of alumina (Al_2_O_3_) were observed. Four diffraction peaks related to monoclinic (Al_2_O_3_) (COD 1200005) were observed at 2θ = 31.2, 36.6, 59.9, and 63.9°, which correspond to the diffraction of (400), (111), (313-), and (020) crystal planes, respectively. The two diffraction peaks at 2θ = 39.9° (222) and 45.7° (040) are related to cubic (Al_2_O_3_) (COD 1101168). The diverse array of compounds found in the UNP sample enhances its catalytic efficiency, which will be elaborated upon in subsequent discussions. Finally, the average crystallite size for the UNP sample was calculated using the Scherrer equation^40^ and found to be 47.53 nm, which indicated that the sample is nanocrystalline.

#### 3.1.3 Fourier Transform Infrared (FT-IR) Analysis

Fourier transform infrared spectroscopy (FTIR) is another vital measurement technique that provides information on the surface’s visible functional groups. The FTIR analysis of the original CKD, as delivered, and the UNP sample calcined at 500 °C is depicted in Figure 1b. The FT-IR spectrum of the original CKD sample displays a collection of bands in the 800–1200 cm^–1^ range, which are linked to the asymmetric and symmetric stretching vibrations of Si-O bonds.^4,41,42^ Similarly, the FT-IR spectrum of the UNP sample exhibits two absorption bands in the same range at 810 and 1100 cm^–1^, as in the original CKD sample’s FT-IR spectrum. However, the FT-IR spectrum of the UNP sample shows a significant increase in the Si-O peaks. The strong intensity band observed at 1100 cm^–1^ is linked to the stretching vibration of the Si-O-Si bonds. The other band at 810 cm^–1^ is due to the stretching vibration mode of O-Si-O bonds.^43,44^ Moreover, the FTIR spectrum of the UNP sample shows a strong, sharp band located at 466 cm^–1^, corresponding to the O-Si-O/Si-O-Si bending mode.^42,44^ The bending vibration of the OH groups of adsorbed water is responsible for the band observed at 1644 cm^–1^ in the original CKD spectrum and at 1640 cm^–1^ in the UNP sample.^44,45^

Additionally, the broad band at 3432 cm^–1^ in both spectra is associated with the stretching vibration of the OH group in H_2_O, particularly in the original CKD sample.^44,45^ As it is impossible to prevent the incorporation of CO_2_ when the sample is exposed to air, the small band at 1440 cm^–1^ in the UNP sample is attributed to the asymmetric stretching (ν3) of CO_3_^2–^. This band appears as a strong absorption band at 1419 cm^–1^ in the original CKD spectrum due to the formation of CaCO_3_ in CKD from atmospheric CO_2._^46^ Finally, a very small, sharp band observed at 1384 cm^–1^ in the UNP sample spectrum is attributed to the vibration of nitrate traces produced during the dissolution of the CKD sample using HNO_3._^4^

#### 3.1.4 Energy-Dispersive X-ray (EDX) and Elemental Mapping Analysis

To identify the elemental compositions or even trace elements in the UNP sample calcined at 500 °C prepared from CKD, energy-dispersive X-ray (EDX) was performed. As can be seen in Figure 1c, the EDX pattern clearly supports the good dispersion of the UNP sample. The recorded results of the EDX analysis for the typical sample exhibited that it was composed of Silicon (Si), Oxygen (O), Calcium (Ca), Iron (Fe), and Aluminum (Al) elements. Additionally, a quantitative EDX analysis was conducted on the chosen area to determine the elemental weights and atomic percentages for characterization. The EDX elemental analysis confirmed peaks for Si, O, Ca, Fe, and Al at respective energies having weight percent of 61.03, 29.06, 7.95, 1.23, and 0.73% and atomic contribution of 74.85, 20.30, 3.89, 0.43, and 0.53%, respectively.

**Figure 3:**
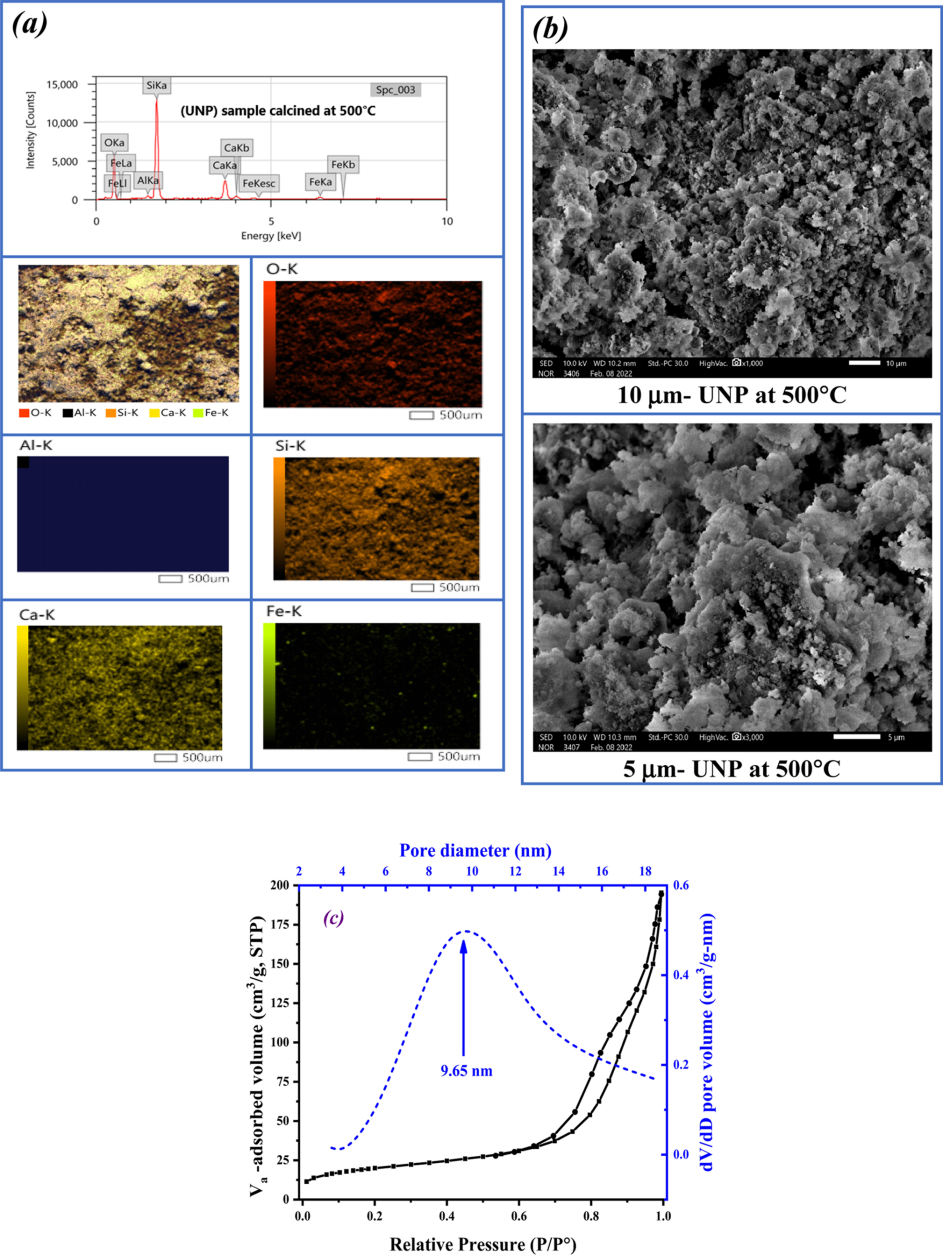
(a) Elemental mapping of UNP sample calcined at 500 °C prepared from CKD for 3 h in an oxygen flow of 100 mL·min^–1^, (b) SEM images of UNP sample calcined at 500 °C prepared from CKD for 3 h in an oxygen flow of 100 mL·min^–1^ with different magnifications, and (c) N_2_ adsorption/desorption isotherm and pore diameter profiles of UNP sample calcined at 500 °C prepared from CKD for 3 h in an oxygen flow of 100 mL·min^–1^.

In order to further illustrate the structural features of the sample, elemental mappings were performed selectively on the UNP sample. The corresponding elemental mapping images are shown in Figure 3a. These images demonstrated the presence of the elements Si, O, Ca, Fe, and Al, which was in accordance with the previous EDX results. Furthermore, red, orange, yellow, green, and black signals in Figure 3a represent silicon (Si), oxygen (O), calcium (Ca), iron (Fe), and aluminum (Al) elements, respectively. The represented elemental mapping present investigation demonstrates that the main constituents of the UNP sample were recorded across the whole spectrum, and the distribution of those components was uniform throughout the sample. Finally, based on the above EDX/elemental mapping analysis, the main elements are Si, O, Ca, and minor amounts of Fe and Al, and these results are in complete agreement with XRD and FT-IR analyses.

#### 3.1.5 Scanning Electron Microscopy (SEM) Analysis

In order to characterize the morphology, topology, and other detailed surface structures of the UNP sample calcined at 500 °C prepared from CKD, scanning electron microscopy (SEM) was utilized. The SEM images of the UNP sample at various magnifications are displayed in Figure 3b. The morphology of the UNP sample shows hierarchical units that are connected to form a coral reef-like shape.^47^ Also, it was found that more drop wells and pores are generated on the surface. Understanding, assigning, and correlating the catalytic performance of the UNP sample when it acts as a catalyst with the porosity, shape, size, distribution, and ordering of the various structures found in such a material all depend on its morphological characteristics. The UNP sample’s unique coral reef-like porous structure demonstrated exceptional catalytic performance by providing abundant binding sites on its surface to enhance the catalytic activity during the catalytic process, which will be covered later.

#### 3.1.6 Surface Area Measurements *S*_BET_

Nitrogen adsorption and desorption isotherms were measured to determine the specific surface area and analyze the pore characteristics of the UNP sample calcined at 500 °C in detail. As illustrated in Figure 3c, the UNP sample exhibits a type IV adsorption–desorption isotherm with an H3-type hysteresis loop according to the classification of the International Union of Pure and Applied Chemistry, which indicates the presence of mesopores (2 nm < size < 50 nm).^48^ Desorption from mesopores typically occurs at pressures lower than those of adsorption, resulting in a hysteresis effect.^49^ Isotherms displaying type H3 hysteresis do not display any apparent adsorption limit at high P/P0. This phenomenon can be attributed to factors like the presence of loosely connected plate-like particle aggregates or configurations of narrow slit-shaped pores, and, in principle, should not be relied upon for an accurate assessment of either the pore size distribution or the overall pore volume.^49,50^ The behavior of the isotherms is influenced by the structure of the pores and the extent of interactions between the adsorbent and the adsorbate system. In simpler terms, the interplay between the adsorbate–adsorbent system, the scale of pore sizes, and the mechanisms of storage are interconnected.^50^ The specific surface area of the UNP sample was determined to be 72.03 m^2^·g^–1^ using the Brunauer–Emmett–Teller (BET) method. The pore diameter was determined with the Barrett–Joyner–Halenda (BJH) method based on the adsorption branch data. The pore size distribution (PSD) curve of the UNP sample shows a broad pore size distribution with a maximum peak of 9.65 nm, which suggests the evident characteristics of the mesoporous nature of the UNP sample. Based on the above data, it can be concluded that the UNP sample possesses a well-organized mesoporous structure. The sample’s high surface area and abundant mesopores are likely to enhance effective contact between active sites on the catalyst surface and facilitate mass transport during the catalytic reaction, which will be discussed later.

#### 3.1.7 X-ray Photoelectron Spectroscopy (XPS) and Transmission Electron Microscopy (TEM) Analysis

**Figure 4:**
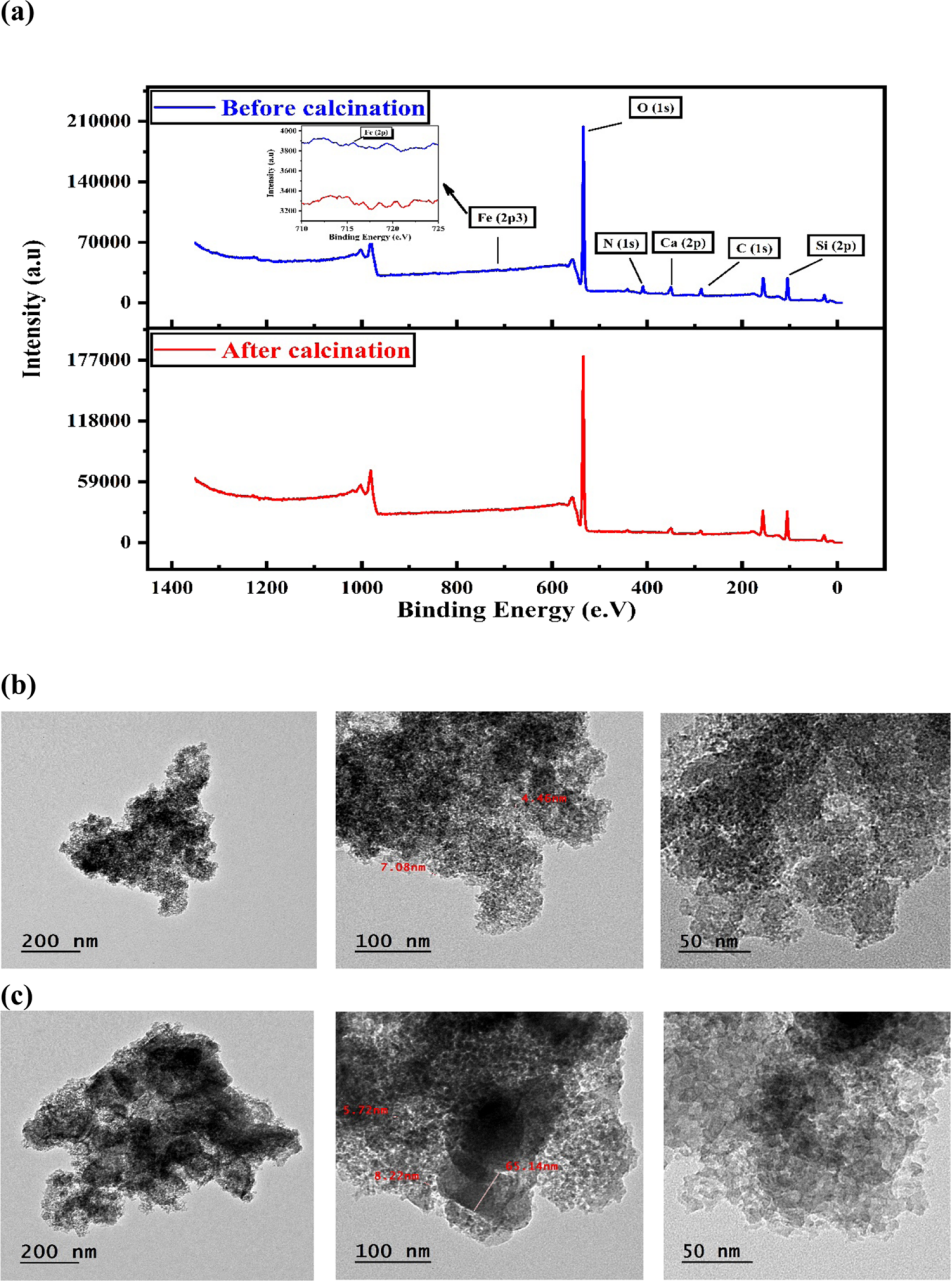
(a) XPS spectra for uncalcined and calcined UNP sample at 500 °C, (b) TEM images for uncalcined UNP sample, and (c) TEM images for calcined UNP sample at 500 °C.

XPS analysis was used to investigate the surface characteristics of the UNP sample before and after calcination. Figure 4a shows the XPS survey of the surface of the UNP sample before and after calcination, which confirms the presence of the claimed elements. As shown in Figure 4a, six XPS peaks can be seen in the case of the noncalcined sample, which were assigned to Si 2p, C 1s, Ca 2p, N 1s, O 1s, and Fe 2p3 from the side of lower binding energy. In the case of the calcined UNP sample, five XPS peaks are observed assigned to Si 2p, C 1s, Ca 2p, O 1s, and Fe 2p3. The disappearance of the N 1s peak in the calcined sample is likely due to the removal of the residual nitrates from the sample surface during the calcination step.

TEM is used to study the topographical, morphological, and particle size information on the UNP sample. Figure 4b shows the TEM images of the noncalcined sample, which reveals irregularly shaped nanoparticles. In addition, the images display varying degrees of darkness, indicating differences in the sample density, which is expected in the case of multicomponent samples. As shown in Figure 4c, the calcined sample has the same irregularly shaped nanoparticles and displays varying degrees of darkness. However, compared to the noncalcined sample, the particle size is slightly increased in the calcined sample, and the particles started to aggregate, forming larger clusters, which is likely due to calcination.

#### 3.1.8 Surface Acidity Measurements

**Figure 5:**
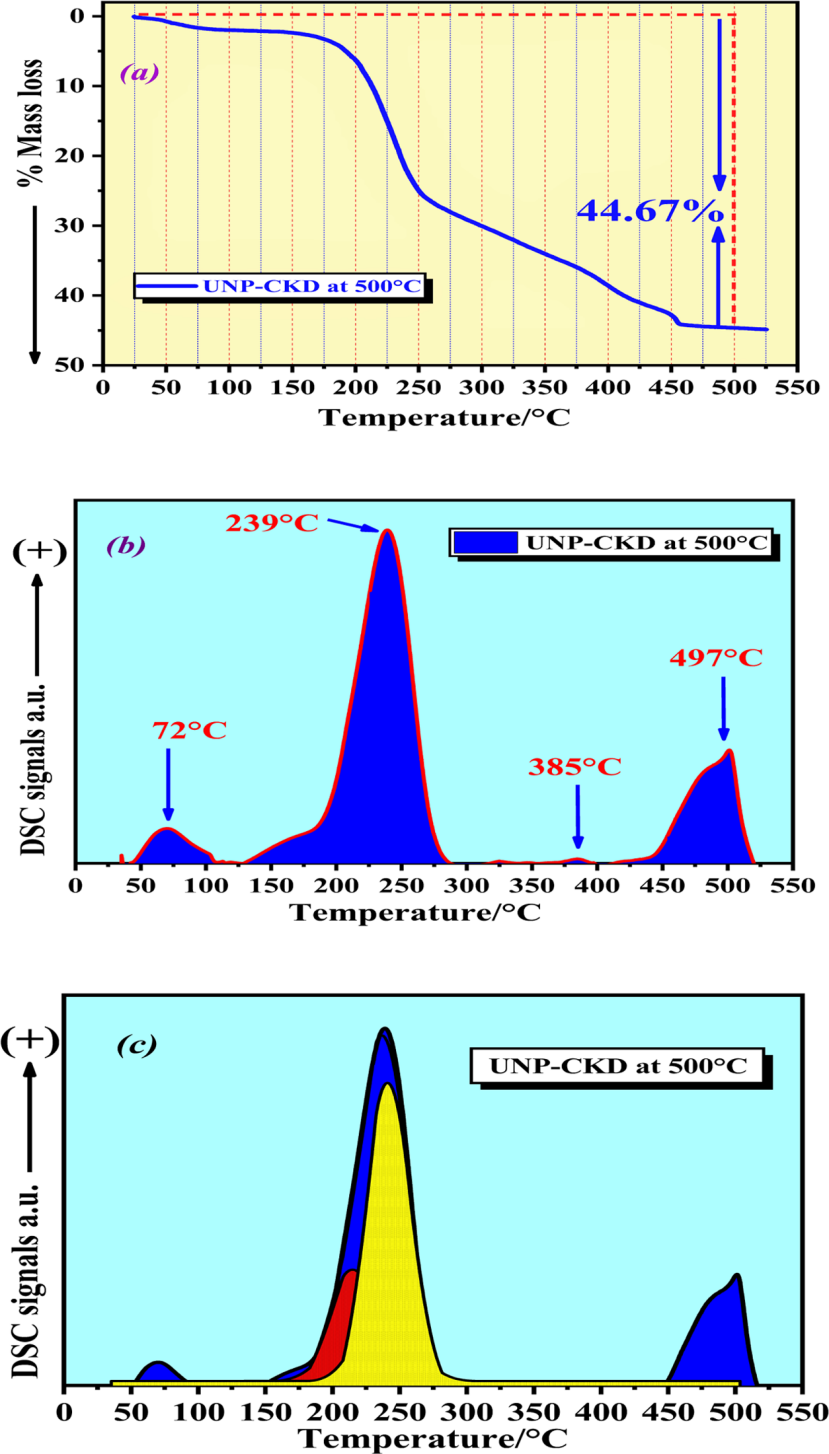
(a) TG-TPD profile of the %mass loss of THF as probe molecule for UNP sample calcined at 500 °C, (b) DSC-TPD profile recorded during desorption of THF as probe molecule for UNP sample calcined at 500 °C, and (c) DSC-TPD profile recorded during desorption of THF as probe molecule as well as the resulting Gaussian deconvolution profile of UNP sample calcined at 500 °C prepared from CKD for 3 h in an oxygen flow of 100 mL·min^–1^.

The distribution of surface acid sites on a UNP sample was analyzed by using temperature-programmed desorption techniques. Specifically, tetrahydrofuran (THF-TPD) was used to investigate the total population and the strength distribution of acidic sites.^34^ TG-TPD of THF was used to determine the total population of acid sites on the sample, and the DSC-TPD technique was employed to monitor the acid sites with varying strengths. As shown in Figure 5a, the TG-TPD curve exhibits a total mass loss of 44.67% due to the desorption of THF molecules in the temperature range of RT-500 °C. This weight loss corresponded to a calculated total number of acidic sites of 3.73 × 10^21^ sites·g^–1^. As shown in Figure 5b, the DSC-TPD curve of the UNP sample revealed important details about the distribution of these acidic sites over the sample surface as different bands at different temperatures correspond to acidic sites with different strengths. As the peak temperature in the case of DSC curve is correlated to the strength of acidic sites, the area under the peak can be correlated to the amount (the number) of the acidic sites.

In Figure 5b, the DSC-TPD profile displayed a small peak at 72 °C that was related to weak acidic sites, a large peak at 239 °C, which represented moderate acidic sites, a very faint peak at 385 °C, and a strong peak at 497 °C, which were attributed to strong acidic sites. To better classify the massive peak at 239 °C associated with moderately acidic sites in the THF-DSC profile, a deconvolution Gaussian line form was used, as shown in Figure 5c. This accurately divided the massive peak into two subacidic sites, as illustrated in Figure 5c. The number of moderately acidic sites calculated under this peak was 2.25 × 10^21^ sites·g^–1^. The wide range of acidic sites with varying strengths over the UNP sample can clarify the superior catalytic activity and selectivity of this sample toward the production of ethylene as the main product in the bioethanol reaction, which will be discussed in the Catalytic Activitysection.

### 3.2 Decomposition of Bioethanol over UNP Catalyst

Bioethanol is a highly promising and significant renewable energy source both from an environmental and economic standpoint.^51^ Among various methods of ethanol conversion, the catalytic conversion of ethanol to ethylene has garnered significant attention. There is a growing demand for the production of ethylene, and as bioethanol conversion to bioethylene is a catalytic process, finding efficient and pristine catalysts has become increasingly challenging. Catalytic dehydration of bioethanol is an alternative method for producing bioethylene. Although the catalytic dehydration of ethanol to ethylene was first reported back in 1797, the first commercial plant was established in the early 20th century.^52^

In order to design a process and evaluate the economic impact and configuration of reaction conditions, it is necessary to assess catalyst performance.^53^ The catalytic performance of the UNP catalyst was studied in the decomposition of bioethanol as a test reaction. The results showed that the UNP catalyst successfully converted bioethanol to ethylene and DEE via a single-step alcohol dehydration process, making it an effective acidic catalyst. The UNP catalyst is considered a multicomponent catalyst, as it contains SiO_2_, CaSiO_3_, Fe_2_O_3_, Fe_2_SiO_4_, and Al_2_O_3_, as shown in XRD analysis. Multicomponent catalysts have long been known to have the potential for improving catalytic performance,^54^ and there has been an increased interest in them recently. This arises from the understanding that the scaling relationships governing the adsorption and transition state energies of reactions impose substantial constraints on the enhancement of catalyst performance through the use of catalysts with a singular function.^55^ The catalytic activity results and catalyst preparation have shown a high level of consistency, and the carbon mass balance was almost 100% in all of the catalytic tests. In order to achieve a cost-effective production of ethylene and DEE from bioethanol in a one-step process, a series of experiments were carried out:-

#### 3.2.1 Effect of Carrier Gas

**Figure 6:**
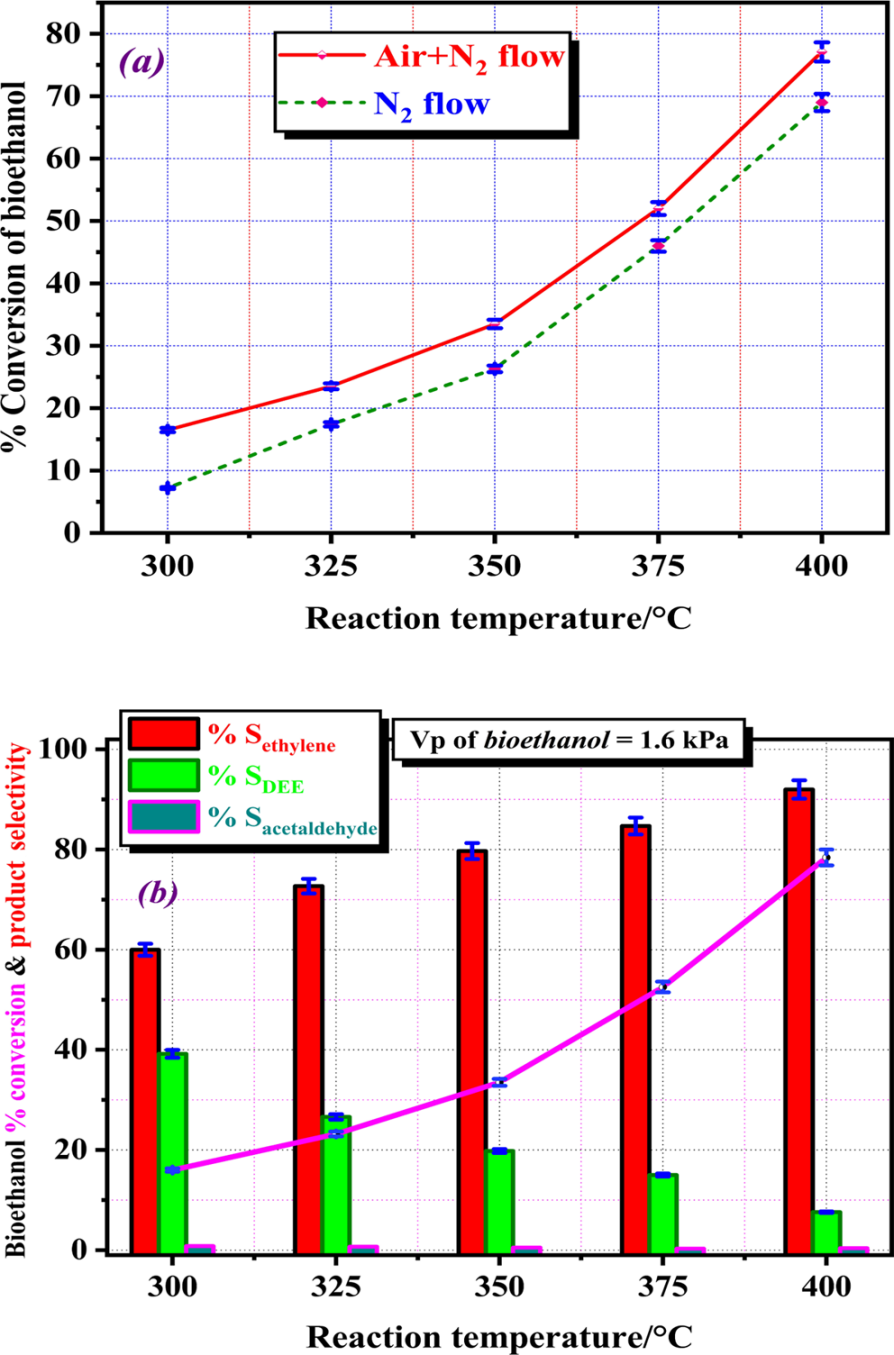
(a) Effect of carrier gas during the conversion of bioethanol over UNP catalyst calcined at 500 °C prepared from CKD for 3 h in an oxygen flow of 100 mL·min^–1^ and (b) %conversion and %selectivity for the catalytic decomposition of bioethanol over UNP catalyst calcined at 500 °C prepared from CKD for 3 h in an oxygen flow of 100 mL·min^–1^. The error bars represent the standard deviations from two trials.

The effect of the carrier gas on the catalytic activity of the UNP catalyst during the dehydration of bioethanol was investigated. The conversion of bioethanol was studied using 0.5 g of UNP catalyst and a Vp of bioethanol of 1.6 kPa in the temperature range of 300–400 °C, with 50 mL·min^–1^ of N_2_ gas or a mixture of N_2_ (25 mL·min^–1^) and air (25 mL·min^–1^) as carriers in two separate experiments with a gas hourly space velocity (GHSV) of 6 L·g_cat_^–1^·h^–1^. As depicted in Figure 6a, there was a higher conversion of bioethanol using the mixture compared with only N_2_ gas at all reaction temperatures. This indicates that incorporating air with N_2_ enhances and activates the active sites on the catalyst surface. The results showed that when a mixture of (N_2_+air) was used as the carrier gas, the %conversion of bioethanol increased by approximately 9.3 and 8.1% at reaction temperatures of 300 and 400 °C, respectively, compared to using N_2_ gas as a carrier. Therefore, the air-nitrogen mixture was used as a carrier in all subsequent experiments.

#### 3.2.2 Effect of Reaction Temperature

To assess their catalytic performance in terms of catalytic activity (%conversion of bioethanol and product selectivity), the catalytic dehydration of bioethanol over UNP catalyst was conducted at various reaction temperatures from 300 to 400 °C. Figure 6b displays the %conversion and the product selectivity during the bioethanol dehydration reaction as a function of reaction temperature. Figure 6b reveals a significant improvement in the catalyst activity by increasing the reaction temperature. The %conversion of bioethanol increased significantly from 16.5% at a reaction temperature of 300 °C to 77.1% at a reaction temperature of 400 °C. Obviously, at lower temperatures, bioethanol dehydration appears to be incomplete, resulting in a poor conversion. In addition, bioethanol conversion over the catalyst under study increases as the reaction temperature rises. This can provide vital information on the surface-active sites, where it becomes more active as the temperature continuously increases up to 400 °C. The selectivity values of the tested catalyst toward the production of ethylene, DEE, and acetaldehyde as dehydration products are depicted in Figure 6b. The obtained results indicated that the main product is ethylene, which increased with increasing reaction temperature. The selectivity to ethylene increased from 60% at 300 °C to 92% at 400 °C. The selectivity to DEE decreased with increasing the reaction temperature, while the selectivity to acetaldehyde was very low at all reaction temperatures applied.

Le Chatelier’s principle states that increasing the temperature in a system favors endothermic reactions, whereas decreasing the temperature favors exothermic reactions. This finding suggests that the dehydration process of ethanol includes an endothermic reaction.^56^ Acidic catalysts are known to produce both ethylene and DEE competitively during the ethanol dehydration process.^57^ The mechanism of this reaction involves two main pathways, which can occur concurrently and sequentially.^58^ According to eq 4, the conversion of one molecule of ethanol requires an endothermic reaction that consumes +45 KJ·mol^–1^ and produces ethylene and water. The second pathway, illustrated in eq 5, is exothermic and releases −25 kJ·mol^–1^ of energy. This pathway involves the reaction of two molecules of ethanol to produce DEE and water. In other words, according to product selectivity, low reaction temperatures are favorable for the formation of DEE via intermolecular dehydration, whereas high reaction temperatures are favorable for the formation of ethylene via intramolecular dehydration.^57,59^
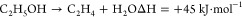
4
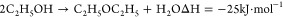
5

**Figure 7:**
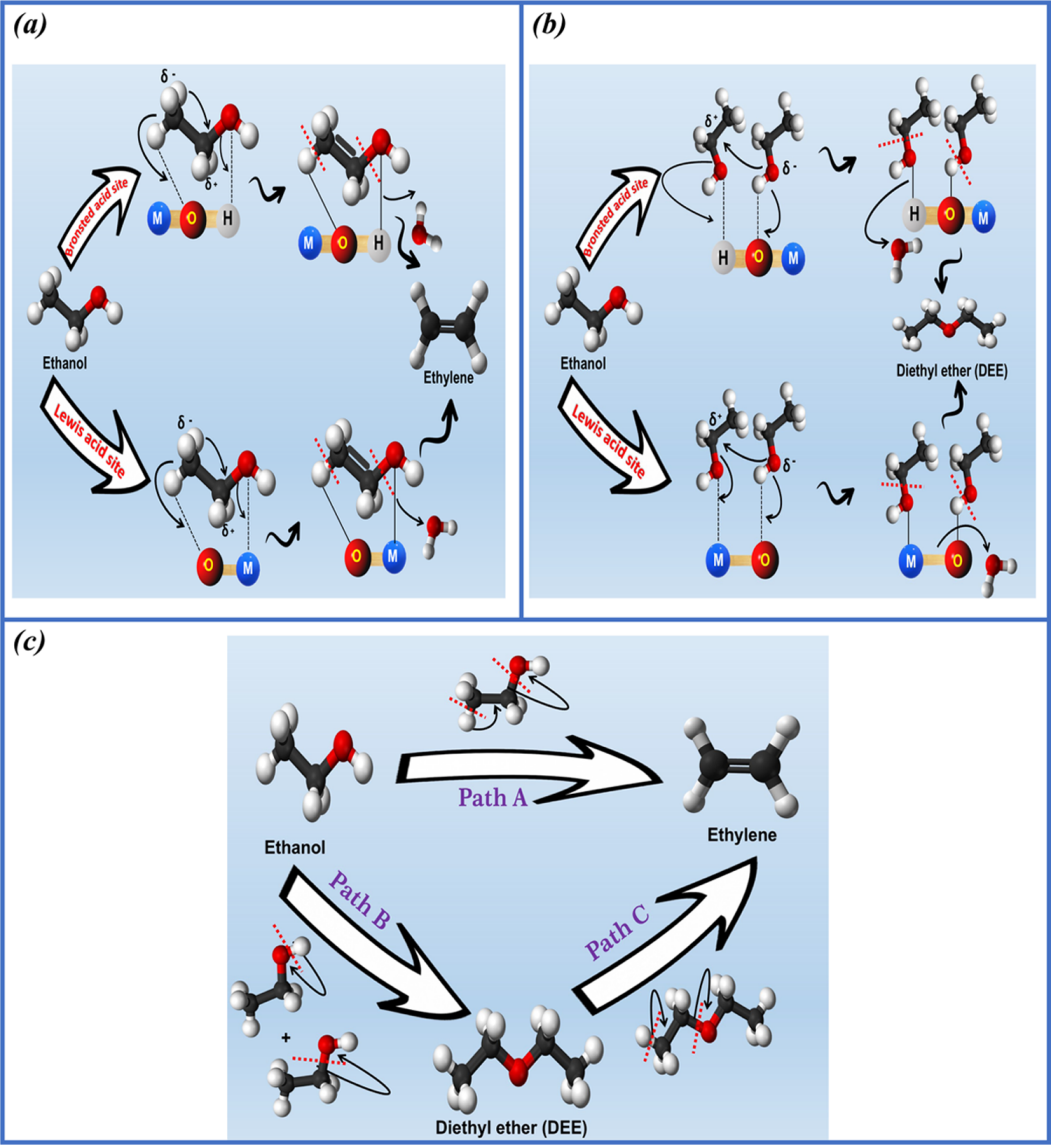
(a) Mechanism of ethylene formation on Brønsted and Lewis acid sites, (b) the mechanism of DEE formation on Brønsted and Lewis acid sites, and (c) reaction pathways for ethanol to ethylene conversion.

The acidity and surface characteristics of catalysts play a crucial role in the efficiency of the dehydration of ethanol to form ethylene and DEE.^59^ Both Lewis and Brønsted acidic sites are involved in the catalytic dehydration of ethanol to produce ethylene and DEE. In this process, the acid catalyst protonates the hydroxyl group, which then leaves as a water molecule, leading to the production of ethylene. The methyl group is then deprotonated by the catalyst’s conjugate base, and the hydrocarbon rearranges into ethylene.^60,61^Figure 7a illustrates the mechanism of ethylene formation on both Lewis and Brønsted acidic sites during the catalytic dehydration of ethanol.

On the other hand, DEE can be produced via two pathways: dissociative or associative. The dissociative process involves ethanol adsorption on the catalyst surface, followed by the elimination of water, resulting in an adsorbed ethyl group. The ethyl group then interacts with the ethanol again, leading to the production of DEE. In the associative pathway, ethanol and DEE coadsorb on the catalyst surface, reacting to form DEE.^61,62^Figure 7b illustrates the mechanism of DEE formation on both Lewis and Brønsted acid sites during the catalytic dehydration of ethanol.

In addition to surface acidity, the surface textural properties of the UNP catalyst can also affect the efficiency of ethanol conversion and ethylene selectivity. A higher specific surface area and pore volume of the UNP catalyst can facilitate the deeper penetration of ethanol reactants, prolong their residence time, and increase the likelihood of side reactions.^31^ Overall, the results suggest that the efficient performance of the UNP catalyst in terms of higher conversion of bioethanol and higher selectivity to ethylene is linked to its high surface area, high surface acidity with varying strengths, and multicomponent nature.

#### 3.2.3 Effect of Weight-Hourly Space Velocity (WHSV) on the Catalyst Reactivity

**Figure 8:**
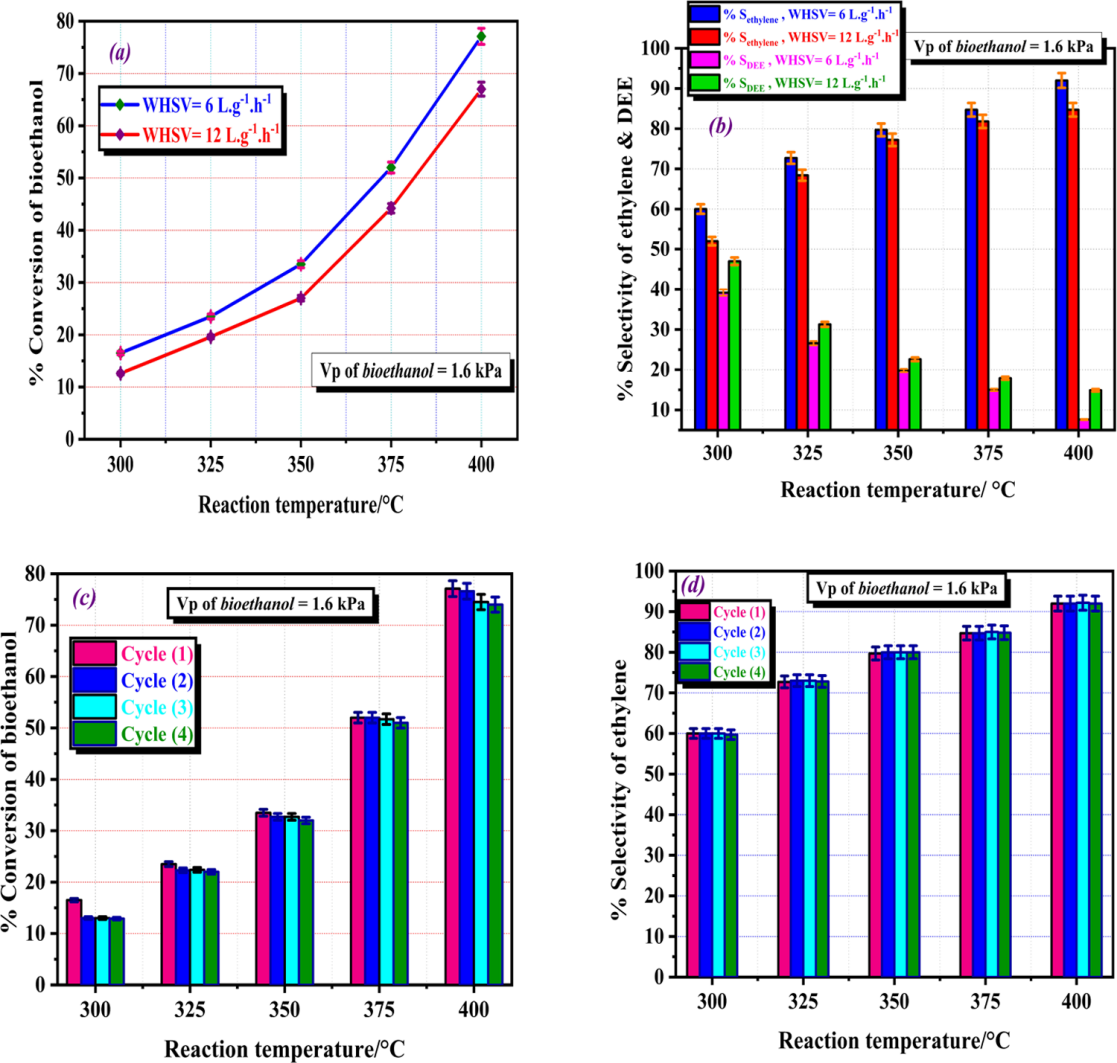
**(a)** Effect of reactant space velocity on the %conversion of bioethanol over UNP catalyst calcined at 500 °C prepared from CKD for 3 h in an oxygen flow of 100 mL·min^–1^, **(b)** effect of reactant space velocity on the catalytic selectivity of ethylene and DEE over the same catalyst, **(c)** reusability and durability of UNP catalyst calcined at 500 °C prepared from CKD for 3 h in an oxygen flow of 100 mL·min^–1^ during the conversion of bioethanol at different reaction temperatures in four successive cycles, and **(d)** selectivity of ethylene in the same four successive cycles. The error bars represent the standard deviation from two trials.

To investigate how space velocity affects the catalytic performance of the UNP catalyst, experiments were conducted using two values of WHSV, i.e., 6 and 12 L·g^–1^·h^–1^. Figure 8a shows the effect of WHSV on bioethanol conversion at temperatures ranging from 300 to 400 °C. The results indicate that higher bioethanol conversion occurred at lower WHSV values. At a reaction temperature of 400 °C, the conversion of ethanol decreased from 77.1 to 67.3% when the value of WHSV increased from 6 to 12 L·g^–1^·h^–1^, see Figure 8a. This finding can be attributed to the fact that a higher space velocity corresponds to a higher flow rate per mass of the catalyst, which reduces the contact time between the reactants and the catalyst. As a result, an increase in space velocity led to a decrease in ethanol conversion.^22,63,64^

The product selectivity during bioethanol dehydration is strongly influenced by WHSV. The same trend of %conversion of bioethanol was observed in the selectivity toward ethylene as the main product. Figure 8b shows that an increase in WHSV from 6 to 12 L·g^–1^·h^–1^ resulted in a reduction of ethylene selectivity from 92 to 84.7% at a reaction temperature of 400 °C. Conversely, the selectivity to DEE increased from 39.2 to 47% with an increase in WHSV from 6 to 12 L·g^–1^·h^–1^ at the same reaction temperature. These observations can be attributed to the elongation of the contact time between bioethanol and active sites at lower WHSV values, resulting in a higher efficiency in intramolecular dehydration.

Conversely, at higher WHSV values, the accumulation of bioethanol promotes the formation of diethyl ether through intermolecular dehydration, leading to lower ethylene selectivity.^64,65^ When a low space velocity of reactant is employed, the reaction pathway is suggested to occur through a parallel series reaction that includes the direct conversion of ethanol into ethylene, ethanol transformation to DEE, and decomposition of DEE to ethylene,^22^ see Figure 7c. Based on these findings, a weight hourly space velocity of 6 L·g^–1^·h^–1^ was used in all experiments that yielded favorable results.

#### 3.2.4 Stability and Reusability of UNP Catalyst

The UNP catalyst studied in this work demonstrated an important feature of stability or the ability to resist deactivation during the bioethanol dehydration into ethylene under operating conditions. In most cases, catalysts are prone to deactivation, which can occur through several mechanisms. This includes the sintering of the active sites, the formation of coke due to the existence of potent acid sites, or the impairment of active acidic sites caused by impurities in the reactant feed.^66^ Therefore, catalyst deactivation is a significant problem that must be considered in this reaction. To confirm the durability and stability of the UNP catalyst, a subsequent four-cycle experiment was conducted at a temperature range of 300–400 °C. The same sample was regenerated for 1 h before each cycle by heating at 420–450 °C in 50 mL·min^–1^ of the air-nitrogen flow. The catalytic activity and selectivity to ethylene remained stable along the four successive cycles, as shown in Figure 8c,d. These findings indicate that the UNP catalyst demonstrated good stability and higher selectivity during the synthesis of bioethylene from bioethanol without deactivation during the four successive cycles. A thorough analysis of the findings unveiled a minimum of three notable benefits: utilization of lower reaction temperatures, achieving high levels of conversion and selectivity toward bioethylene, and maintaining exceptional long-term stability over the course of four consecutive cycles.

In conclusion, UNP of CKD shows significant promise as a multicomponent catalyst for bioethylene production, boasting remarkable performance in yield and selectivity. Its scalability, leveraging abundant CKD from the cement industry, positions it advantageously for large-scale production. The catalyst’s cost-effectiveness, utilizing industrial waste, presents a sustainable waste management solution. Its seamless integration into existing bioethylene production methods further underscores its industrial suitability. Altogether, these attributes mark the UNP catalyst as a viable, environmentally sustainable option for large-scale bioethylene production, showcasing potential in various industrial applications and offering a solution for sustainable waste utilization in the cement industry.

## 4 Conclusions

The study provides a solution to two significant problems facing society by converting hazardous industrial waste into value-added products that can be used as fuel alternatives. By repurposing CKD as a versatile catalyst, the research addresses the energy crisis while promoting global sustainability goals. It effectively tackles waste management and contributes to a greener future. The UNP of the CKD sample was thermally treated at 500 °C in an oxygen flow of 100 mL·min^–1^. UNP sample was characterized using various analysis techniques. According to XRD, EDX, and elemental mapping analysis, the UNP sample consisted of five different compounds. Furthermore, N_2_ adsorption–desorption isotherm indicates that the UNP catalyst is mesoporous with a high BET surface area of 72.03 m^2^·g^–1^ and a pore size of 9.65 nm. TG and DSC-TPD experiments using tetrahydrofuran (THF) as a probe molecule demonstrated the existence of different acidic sites over the UNP catalyst with varying strength. The prepared UNP catalyst was examined during the direct production of ethylene by using the preferential dehydration of bioethanol. Due to the fact that the UNP sample is composed of five different compounds, it can be considered a multicomponent catalyst. The diverse array of compounds found in the UNP sample enhances its catalytic efficiency. Thus, the presence of a large population of acidic sites and high surface area are believed to play a crucial role in catalytic reactions, as they can facilitate the adsorption and activation of reactant molecules. The presence of a large population of acidic sites suggests that the UNP sample may have promising catalytic properties. All of these distinctive characteristics of the UNP sample make it a pristine and promising catalyst. Finally, in the four successive cycles of ethylene synthesis from bioethanol, the UNP catalyst showed good stability and higher selectivity without deactivation. This indicates that the UNP catalyst is highly promising for large-scale industrial applications. Its ability to maintain stability and selectivity over multiple cycles makes it a cost-effective and efficient option for the synthesis of ethylene from bioethanol. By utilizing UNP from CKD, harmful emissions decrease, mitigating environmental impact, and reducing hazardous waste accumulation. Consequently, this research not only supports sustainable development but also presents a valuable contribution to global efforts, harmonizing economic growth with environmental responsibility.

Future research will prioritize scaling up production and assessing the catalyst’s industrial feasibility. Pilot experiments should address challenges such as reactor design, catalyst stability, and process optimization. Conducting a comprehensive life cycle assessment (LCA) will analyze the environmental impact, including energy use, emissions, and waste. This assessment guides greener alternatives by identifying environmental hotspots. Exploring the UNP catalyst’s potential in renewable chemicals beyond its current application can broaden its usage and contribute to a circular economy. Investigating its efficacy in synthesizing diverse biobased polymers or chemicals can significantly expand its applications.

## Supporting Information Available

Table detailing the chemical composition of CKD from Misr Cement-Qena Factory (expressed as weight percentage) determined via X-ray fluorescence (XRF); characterization techniques used: X-ray diffraction (XRD); Fourier transform infrared spectroscopy (FTIR); thermal analyses; surface area measurements; scanning electron microscopy (SEM); energy-dispersive X-ray spectroscopy (EDX); high-resolution transmission electron microscopy (HR-TEM); and X-ray photoelectron spectroscopy (XPS) (PDF)

## References

[ref1] Tong R.; Sui T. B.; Feng L. Z.; Lin L. The digitization work of cement plant in China. Cem. Concr. Res. 2023, 173, 107266 10.1016/j.cemconres.2023.107266.

[ref2] Schneider M. The cement industry on the way to a low-carbon future. Cem. Concr. Res. 2019, 124, 105792 10.1016/j.cemconres.2019.105792.

[ref3] Zheng J.; Du W.; Lang Z.; Qian F. Modeling and Optimization of the Cement Calcination Process for Reducing NOx Emission Using an Improved Just-In-Time Gaussian Mixture Regression. Ind. Eng. Chem. Res. 2020, 59 (11), 4987–4999. 10.1021/acs.iecr.9b05207.

[ref4] Nasr M.; Halawy S. A.; El-Nahas S.; Abdelkader A.; Osman A. I. Direct and easily prepared nanocomposite impurity-free hydroxyapatite derived from CKD as an effective catalyst for trans-2-butene production. Appl. Catal., A 2023, 652, 119039 10.1016/j.apcata.2023.119039.

[ref5] Chaunsali P.; Peethamparan S. Influence of the composition of cement kiln dust on its interaction with fly ash and slag. Cem. Concr. Res. 2013, 54, 106–113. 10.1016/j.cemconres.2013.09.001.

[ref6] El-Attar M. M.; Sadek D. M.; Salah A. M. Recycling of high volumes of cement kiln dust in bricks industry. J. Clean. Prod. 2017, 143, 506–515. 10.1016/j.jclepro.2016.12.082.

[ref7] Drack J. M. E.; Vazquez D. P. Morphological response of a cactus to cement dust pollution. Ecotoxicol. Environ. Saf. 2018, 148, 571–577. 10.1016/j.ecoenv.2017.10.046. 29127819

[ref8] McSorley K.; Rutter A.; Cumming R.; Zeeb B. A. Phytoextraction of chloride from a cement kiln dust (CKD) contaminated landfill with Phragmites australis. Waste Manag. 2016, 51, 111–118. 10.1016/j.wasman.2015.11.009. 26597371

[ref9] Fadhil T. H.; Jasim S. S.; Aziz K. E.; Ahmed A. S. Influence of using White cement kiln dust as a mineral filler on hot asphalt concrete mixture properties. Int. J. Civ. Eng. 2013, 4 (1), 87–96. 10.37649/aengs.2017.130885.

[ref10] Sutter L. L.; Hooton R. D. Progress towards sustainability through performance-based standards and specifications. Cem. Concr. Res. 2023, 174, 107303 10.1016/j.cemconres.2023.107303.

[ref11] Halawy S. A.; Osman A. I.; Nasr M.; Rooney D. W. Mg-O-F Nanocomposite Catalysts Defend against Global Warming via the Efficient, Dynamic, and Rapid Capture of CO_2_ at Different Temperatures under Ambient Pressure. ACS Omega 2022, 7 (43), 38856–38868. 10.1021/acsomega.2c04587. 36340116 PMC9631741

[ref12] Pacewska B.; Wilinska I. Usage of supplementary cementitious materials: advantages and limitations Part I. C-S-H, C-A-S-H and other products formed in different binding mixtures. J. Therm. Anal. Calorim. 2020, 142 (1), 371–393. 10.1007/s10973-020-09907-1.

[ref13] Varjani S.; Shahbeig H.; Popat K.; Patel Z.; Vyas S.; Shah A. V.; Barcelo D.; Ngo H. H.; Sonne C.; Lam S. S.; et al. Sustainable management of municipal solid waste through waste-to-energy technologies. Bioresour. Technol. 2022, 355, 127247 10.1016/j.biortech.2022.127247. 35490955

[ref14] Maliha A.; Abu-Hijleh B. A review on the current status and post-pandemic prospects of third-generation biofuels. Energy Syst. 2022, 1–32. 10.1007/s12667-022-00514-7.

[ref15] Zakeri B.; Paulavets K.; Barreto-Gomez L.; Echeverri L.; Pachauri S.; Boza-Kiss B.; Zimm C.; Rogelj J.; Creutzig F.; Ürge-Vorsatz D. Pandemic, War, and Global Energy Transitions. Energies 2022, 15, 6114 10.3390/en15176114.

[ref16] Guan Y.; Yan J.; Shan Y.; Zhou Y.; Hang Y.; Li R.; Liu Y.; Liu B.; Nie Q.; Bruckner B.; et al. Burden of the global energy price crisis on households. Nat. Energy 2023, 8 (3), 304–316. 10.1038/s41560-023-01209-8.

[ref17] Hoang A. T.; Pandey A.; Huang Z.; Luque R.; Ng K. H.; Papadopoulos A. M.; Chen W.-H.; Rajamohan S.; Hadiyanto H.; Nguyen X. P.; Pham V. V. Catalyst-Based Synthesis of 2,5-Dimethylfuran from Carbohydrates as a Sustainable Biofuel Production Route. ACS Sustainable Chem. Eng. 2022, 10 (10), 3079–3115. 10.1021/acssuschemeng.1c06363.

[ref18] Tutak M.; Brodny J. Renewable energy consumption in economic sectors in the EU-27. The impact on economics, environment and conventional energy sources. A 20-year perspective. J. Clean. Prod. 2022, 345, 131076 10.1016/j.jclepro.2022.131076.

[ref19] Yu D.; Guo J.; Meng J.; Sun T. Biofuel production by hydro-thermal liquefaction of municipal solid waste: Process characterization and optimization. Chemosphere 2023, 328, 138606 10.1016/j.chemosphere.2023.138606. 37023903

[ref20] Wang M.; Qiao J.; Sheng Y.; Wei J.; Cui H.; Li X.; Yue G. Bioconversion of corn fiber to bioethanol: Status and perspectives. Waste Manag. 2023, 157, 256–268. 10.1016/j.wasman.2022.12.026. 36577277

[ref21] Rossetti I.; Tripodi A.; Ramis G. Hydrogen, ethylene and power production from bioethanol: Ready for the renewable market?. Int. J. Hydrogen Energy 2020, 45 (17), 10292–10303. 10.1016/j.ijhydene.2019.07.201.

[ref22] Shetsiri S.; Thivasasith A.; Saenluang K.; Wannapakdee W.; Salakhum S.; Wetchasat P.; Nokbin S.; Limtrakul J.; Wattanakit C. Sustainable production of ethylene from bioethanol over hierarchical ZSM-5 nanosheets. Sustainable Energy Fuels 2019, 3 (1), 115–126. 10.1039/C8SE00392K.

[ref23] Bisztyga-Szklarz M.; Mech K.; Marzec M.; Kalendarev R.; Szacilowski K. In Situ Regeneration of Copper-Coated Gas Diffusion Electrodes for Electroreduction of CO_2_ to Ethylene. Materials 2021, 14 (12), 3171 10.3390/ma14123171. 34207638 PMC8228262

[ref24] Zhang M. H.; Yu Y. Z. Dehydration of Ethanol to Ethylene. Ind. Eng. Chem. Res. 2013, 52 (28), 9505–9514. 10.1021/ie401157c.

[ref25] Mendieta C. M.; Vallejos M. E.; Felissia F. E.; Chinga-Carrasco G.; Area M. C. Review: Bio-polyethylene from Wood Wastes. J. Polym. Environ. 2020, 28 (1), 1–16. 10.1007/s10924-019-01582-0.

[ref26] Salcedo A.; Poggio-Fraccari E.; Marino F.; Irigoyen B. Tuning the selectivity of cerium oxide for ethanol dehydration to ethylene. Appl. Surf. Sci. 2022, 599, 153963 10.1016/j.apsusc.2022.153963.

[ref27] Ozden A.; Wang Y. H.; Li F. W.; Luo M. C.; Sisler J.; Thevenon A.; Rosas-Hernandez A.; Burdyny T.; Lum Y. W.; Yadegari H.; et al. Cascade CO_2_ electroreduction enables efficient carbonate-free production of ethylene. Joule 2021, 5 (3), 706–719. 10.1016/j.joule.2021.01.007.

[ref28] Phung T. K.; Busca G.; Chemicals P. Selective bioethanol conversion to chemicals and fuels via advanced catalytic approaches. In Biorefinery of Alternative Resources: Targeting Green Fuels and Platform Chemicals, 2020, pp 75–103.

[ref29] Anekwe I. M. S.; Isa Y. M.; Oboirien B. Bioethanol as a potential eco-friendlier feedstock for catalytic production of fuels and petrochemicals. J. Chem. Technol. Biotechnol. 2023, 98, 2077 10.1002/jctb.7399.

[ref30] Gao Y. F.; Neal L.; Ding D.; Wu W.; Baroi C.; Gaffney A. M.; Li F. X. Recent Advances in Intensified Ethylene Production-A Review. ACS Catal. 2019, 9 (9), 8592–8621. 10.1021/acscatal.9b02922.

[ref31] Cheng Y. W.; Chong C. C.; Cheng C. K.; Ng K. H.; Witoon T.; Juan J. C. Ethylene production from ethanol dehydration over mesoporous SBA-15 catalyst derived from palm oil clinker waste. J. Clean. Prod. 2020, 249, 119323 10.1016/j.jclepro.2019.119323.

[ref32] Clemente M. C. H.; Martins G. A. V.; de Freitas E. F.; Dias J. A.; Dias S. C. L. Ethylene production via catalytic ethanol dehydration by 12-tungstophosphoric acid@ceria-zirconia. Fuel 2019, 239, 491–501. 10.1016/j.fuel.2018.11.026.

[ref33] Sezer I. A review study on using diethyl ether in diesel engines: Effects on fuel properties, injection, and combustion characteristics. Energy Environ. 2020, 31 (2), 179–214. 10.1177/0958305X19856751.

[ref34] Halawy S. A.; Osman A. I.; Abdelkader A.; Nasr M.; Rooney D. W. Assessment of Lewis-Acidic Surface Sites Using Tetrahydrofuran as a Suitable and Smart Probe Molecule. ChemistryOpen 2022, 11 (3), e202200021 10.1002/open.202200021. 35324079 PMC8944219

[ref35] Osman A. I.; Abu-Dahrieh J. K.; Rooney D. W.; Halawy S. A.; Mohamed M. A.; Abdelkader A. Effect of precursor on the performance of alumina for the dehydration of methanol to dimethyl ether. Appl. Catal., B 2012, 127, 307–315. 10.1016/j.apcatb.2012.08.033.

[ref36] Tavares L. R. C.; Junior J. F. T.; Costa L. M.; da Silva Bezerra A. C.; Cetlin P. R.; Aguilar M. T. P. Influence of quartz powder and silica fume on the performance of Portland cement. Sci. Rep. 2020, 10 (1), 21461 10.1038/s41598-020-78567-w. 33293621 PMC7722713

[ref37] Hong J.; Yang F.; Sun Z. Hexagonal bi-pyramid α-Fe2O3 microcrystals: Unusual formation, characterization and application for gas sensing. J. Alloys Compd. 2021, 889, 161515 10.1016/j.jallcom.2021.161515.

[ref38] Amin A. M. M.; El-Amir A. A. M.; Karunakaran G.; Kuznetsov D.; Ewais E. M. M. In-vitro evaluation of wollastonite nanopowder produced by a facile process using cheap precursors for biomedical applications. Ceram. Int. 2021, 47 (13), 18684–18692. 10.1016/j.ceramint.2021.03.201.

[ref39] Guo P. S.; Wang C. X. Good lithium storage performance of Fe_2_SiO_4_ as an anode material for secondary lithium ion batteries. Rsc Adv. 2017, 7 (8), 4437–4443. 10.1039/C6RA26376C.

[ref40] Holzwarth U.; Gibson N. The Scherrer equation versus the ’Debye-Scherrer equation’. Nat. Nanotechnol. 2011, 6 (9), 534 10.1038/nnano.2011.145. 21873991

[ref41] Ashraf W.; Olek J. Carbonation behavior of hydraulic and non-hydraulic calcium silicates: potential of utilizing low-lime calcium silicates in cement-based materials. J. Mater. Sci. 2016, 51 (13), 6173–6191. 10.1007/s10853-016-9909-4.

[ref42] Giraudo N.; Bergdolt S.; Wohlgemuth J.; Welle A.; Schuhmann R.; Koniger F.; Thissen P. Calcium Silicate Phases Explained by High-Temperature-Resistant Phosphate Probe Molecules. Langmuir 2016, 32 (51), 13577–13584. 10.1021/acs.langmuir.6b03218. 27973852

[ref43] Gabor A. E.; Davidescu C. M.; Negrea A.; Ciopec M.; Butnariu M.; Ianasi C.; Muntean C.; Negrea P. Lanthanum Separation from Aqueous Solutions Using Magnesium Silicate Functionalized with Tetrabutylammonium Dihydrogen Phosphate. J. Chem. Eng. Data 2016, 61 (1), 535–542. 10.1021/acs.jced.5b00687.

[ref44] Lee B. S.; Lin H. P.; Chan J. C.; Wang W. C.; Hung P. H.; Tsai Y. H.; Lee Y. L. A novel sol-gel-derived calcium silicate cement with short setting time for application in endodontic repair of perforations. Int. J. Nanomed. 2018, Volume 13, 261–271. 10.2147/IJN.S150198. PMC5764294 29386894

[ref45] de Mendonça E. S. D. T.; de Faria A. C. B.; Dias S. C. L.; Aragón F. F. H.; Mantilla J. C.; Coaquira J. A. H.; Dias J. A. Effects of silica coating on the magnetic properties of magnetite nanoparticles. Surfaces and Interfaces 2019, 14, 34–43. 10.1016/j.surfin.2018.11.005.

[ref46] Liu L. F.; Xu Z. G.; Liu Y.; Yin Z. Z.; Sheng Y. S.; Ding C. Q.; Kong Y. Facile synthesis of calcium carbonate/polyacrylic acid hydrogels for pH-responsive delivery of cytarabine. J Saudi Chem Soc 2021, 25 (11), 101344 10.1016/j.jscs.2021.101344.

[ref47] Ma S. L.; Tan Y. S.; Han Y. Z. Methanation of syngas over coral reef-like Ni/Al_2_O_3_ catalysts. J. Nat. Gas Chem. 2011, 20 (4), 435–440. 10.1016/S1003-9953(10)60192-2.

[ref48] Groen J. C.; Peffer L. A. A.; Perez-Ramirez J. Pore size determination in modified micro- and mesoporous materials. Pitfalls and limitations in gas adsorption data analysis. Microporous Mesoporous Mater. 2003, 60 (1–3), 1–17. 10.1016/S1387-1811(03)00339-1.

[ref49] Abdelkader A.; Osman A. I.; Halawy S. A.; Mohamed M. A. Preparation and characterization of mesoporous γ-Al_2_O_3_ recovered from aluminum cans waste and its use in the dehydration of methanol to dimethyl ether. J. Mater. Cycles Waste Manage. 2018, 20 (3), 1428–1436. 10.1007/s10163-018-0702-0.

[ref50] Thommes M. Physical Adsorption Characterization of Nanoporous Materials. Chem.-Ing.-Tech. 2010, 82 (7), 1059–1073. 10.1002/cite.201000064.

[ref51] Liu C. Y.; Struwe K.; Lee C. H.; Chuang H. Y.; Sauer J.; Yu J. C. C.; Nguyen V. H.; Huang C. W.; Wu J. C. S. Ethanol conversion to selective high-value hydrocarbons over Ni/HZSM-5 zeolite catalyst. Catal. Commun. 2020, 144, 106067 10.1016/j.catcom.2020.106067.

[ref52] Mohsenzadeh A.; Zamani A.; Taherzadeh M. J. Bioethylene Production from Ethanol: A Review and Techno-economical Evaluation. Chembioeng. Rev. 2017, 4 (2), 75–91. 10.1002/cben.201600025.

[ref53] Cabello González G.; Concepción P.; Villanueva Perales A. L.; Martínez A.; Campoy M.; Vidal-Barrero F. Ethanol conversion into 1,3-butadiene over a mixed Hf-Zn catalyst: Effect of reaction conditions and water content in ethanol. Fuel Process. Technol. 2019, 193, 263–272. 10.1016/j.fuproc.2019.04.036.

[ref54] Chen Y. L.; Lin J.; Chen X. H.; Fan S. Q.; Zheng Y. Engineering multicomponent metal-oxide units for efficient methane combustion over palladium-based catalysts. Catal. Sci. Technol. 2021, 11 (1), 152–161. 10.1039/D0CY01742F.

[ref55] Kumar G.; Nikolla E.; Linic S.; Medlin J. W.; Janik M. J. Multicomponent Catalysts: Limitations and Prospects. ACS Catal. 2018, 8 (4), 3202–3208. 10.1021/acscatal.8b00145.

[ref56] Chaichana E.; Boonsinvarothai N.; Chitpong N.; Jongsomjit B. Catalytic dehydration of ethanol to ethylene and diethyl ether over alumina catalysts containing different phases with boron modification. J. Porous Mater. 2019, 26 (2), 599–610. 10.1007/s10934-018-0663-7.

[ref57] Wu C. Y.; Wu H. S. Ethylene Formation from Ethanol Dehydration Using ZSM-5 Catalyst. ACS Omega 2017, 2 (8), 4287–4296. 10.1021/acsomega.7b00680. 31457720 PMC6641935

[ref58] Masih D.; Rohani S.; Kondo J. N.; Tatsumi T. Catalytic dehydration of ethanol-to-ethylene over Rho zeolite under mild reaction conditions. Microporous Mesoporous Mater. 2019, 282, 91–99. 10.1016/j.micromeso.2019.01.035.

[ref59] Autthanit C.; Likitpiriya N.; Praserthdam P.; Jongsomjit B. Development of a New Ternary Al_2_O_3_-HAP-Pd Catalyst for Diethyl Ether and Ethylene Production Using the Preferential Dehydration of Ethanol. ACS Omega 2021, 6 (30), 19911–19923. 10.1021/acsomega.1c02818. 34368578 PMC8340412

[ref60] Eagan N. M.; Kumbhalkar M. D.; Buchanan J. S.; Dumesic J. A.; Huber G. W. Chemistries and processes for the conversion of ethanol into middle-distillate fuels. Nat. Rev. Chem. 2019, 3 (4), 223–249. 10.1038/s41570-019-0084-4.

[ref61] Wu X.; Fang G.; Tong Y.; Jiang D.; Liang Z.; Leng W.; Liu L.; Tu P.; Wang H.; Ni J.; Li X. Catalytic Upgrading of Ethanol to n-Butanol: Progress in Catalyst Development. ChemSusChem 2018, 11 (1), 71–85. 10.1002/cssc.201701590. 28895302

[ref62] Akti F. Effect of kaolin on aluminum loading success in synthesis of Al-SBA-15 catalysts: Activity test in ethanol dehydration reaction. Microporous Mesoporous Mater. 2020, 294, 109894 10.1016/j.micromeso.2019.109894.

[ref63] Portillo Crespo M. A.; Vidal-Barrero F.; Azancot L.; Reina T. R.; Campoy M. Insights on Guerbet Reaction: Production of Biobutanol From Bioethanol Over a Mg-Al Spinel Catalyst. Front. Chem. 2022, 10, 945596 10.3389/fchem.2022.945596. 35910746 PMC9329697

[ref64] Yao J.; Liu S.; Chen G.; Yi W.; Liu J. Enhanced bioethanol-to-ethylene performance over nanosized sheet-like M-SAPO-34 (M = Sr and K) catalysts. Microporous Mesoporous Mater. 2022, 338, 111980 10.1016/j.micromeso.2022.111980.

[ref65] Vondrová P.; Tisler Z.; Kocik J.; Carmona H. D.; Murat M. Comparison of doped ZSM-5 and ferrierite catalysts in the dehydration of bioethanol to ethylene in a flow reactor. React. Kinet., Mech. Catal. 2021, 132 (1), 449–462. 10.1007/s11144-021-01925-w.

[ref66] Said A. E.-A. A.; Goda M. N.; Kassem M. A. Promotional Effect of B_2_O_3_, WO_3_ and ZrO_2_ on the Structural, Textural and Catalytic Properties of FePO_4_ Catalyst Towards the Selective Dehydration of Methanol into Dimethyl Ether. Catal. Lett. 2020, 150 (6), 1714–1728. 10.1007/s10562-019-03081-2.

