# Supplementary material for: Utilizing Undissolved Portion (UNP) of Cement Kiln Dust as a Versatile Multicomponent Catalyst for Bioethylene Production from Bioethanol: An Innovative Approach to Address the Energy Crisis
Source: ACS Omega. 2023 Dec 28;9(1):1962–76. doi: 10.1021/acsomega.3c09043 (PMC10785308; doi:10.1021/acsomega.3c09043)
Supplement: Supplementary file 1 — ao3c09043_si_001.pdf [file ao3c09043_si_001.pdf]

Supporting Information

Utilizing Undissolved Portion (UNP) of Cement Kiln Dust as a Versatile Multicomponent Catalyst for Bioethylene Production from Bioethanol: An Innovative Approach to Address the Energy Crisis

Mahmoud Nasr[a], Adel Abdelkader[a], Safaa El-Nahas[a], Ahmed I. Osman[a,b*] , Amal Abdelhaleem[c], Hossam AbdelFattah El Nazer[d], David W. Rooney[b], Samih A. Halawy[a]

[a] Nanocomposite Catalysts Lab., Chemistry Department, Faculty of Science at Qena, South Valley University, Qena 83523, Egypt.

[b] School of Chemistry and Chemical Engineering, Queen's University Belfast, Belfast BT9 5AG, Northern Ireland, UK.

[c] Environmental Engineering Department, Egypt-Japan University of Science and Technology (E-JUST), Alexandria, 21934, Egypt.

[d] Photochemistry Department, National Research Center, Dokki, Giza 12622, Egypt.

* Corresponding author.

Ahmed I. Osman; Email: aosmanahmed01@qub.ac.uk

Address: School of Chemistry and Chemical Engineering, Queen's University Belfast, David Keir Building, Stranmillis Road, Belfast BT9 5AG, Northern Ireland, United Kingdom.

Fax: +44 2890 97 4687

Tel.: +44 2890 97 4412

Table S1. The chemical composition of CKD obtained from Misr Cement-Qena Factory, expressed as weight percentage, was determined using x-ray fluorescence (XRF)

**Wt.%**	**63.14 ± 0.25**	**13.7 ± 0.32**	**5.29 ± 0.07**	**3.38 ± 0.14**	**0.66 ± 0.01**	**4.06 ± 0.26**	**1.13 ± 0.03**	**0.9 ± 0.04**
**Compound or element**	**CaO**	**SiO_2**	**Al_2O_3**	**Fe_2O_3**	**MgO**	**SO_3**	**K_2O**	**Cl**

Characterization Techniques

The X-ray powder diffraction (XRD) analysis of the original CKD sample and the calcined samples at 500°C was conducted using a Brucker AXS-D8 Advance diffractometer (Germany). The instrument was equipped with a copper anode that generated Ni-filtered CuKa radiation (k = 1.5406 Å) from a generator that operated at 40 kV and 40 mA, and the analysis was conducted in the 2θ range between 20°-80°. The DIFFRACplus SEARCH and DIFFRACplus EVA interfaces were utilized to automatically search and match the crystalline phases with the COD crystallographic database for identification purposes.

The FTIR spectra were obtained using a Magna-FTIR 500 (USA) with Nicolet Omnic software and the KBr disk technique, and the spectra were recorded between 4000 and 250 cm^{-1}.

The prepared UNP sample was subjected to thermal analyses using thermogravimetric (TG) and differential scanning calorimetric (DSC) techniques. The experiments were conducted with a heating rate of 10°C.min^{-1} in 40 mL.min^{-1} N_2-gas flow rate, using a 50H Shimadzu thermal analyzer-Japan. The instrument was equipped with a data acquisition and handling system (TA-50WSI), and highly sintered α–Al_2O_3 was used as a reference material in the DSC experiments.

The surface textural properties, including the specific surface area, pore volume, and mean pore radius of the UNP sample calcined at 500°C were determined

by analyzing nitrogen adsorption-desorption isotherms recorded at a temperature of liquid nitrogen (-196°C). This analysis was carried out using an automatic Micromeritics ASAP2010 (U.S.) equipped with online data acquisition and handling system operating BET and BJH analytical software. Prior to the measurements, all the samples were degassed at 200°C and 10^{-5} Torr for two hours. (1 Torr = 133.3 Pa).

The morphological characterization of the UNP sample was performed using Scanning Electron Microscopy (SEM) with a FEI Quanta 250 FEG MKII, which had a high-resolution environmental microscope (ESEM) and XT microscope Control software. EDX dot mapping analysis combined with FESEM was employed to determine the homogeneous dispersion of sample constituents. SEM images of the UNP sample were obtained using a FEI Quanta 250 FEG MKII with a high-resolution environmental microscope (ESEM) and XT Microscope Control software linked to an Electron Dispersive X-ray (EDX) detector. The EDX detector that was used was a 10 mm^2 SDD Detector-x-act from Oxford Instruments, which utilized Aztec® EDX analysis software.

The HR-TEM images were obtained using a JOEL JEM-HR-2100 electron microscope with 200 KV accelerating voltage and 400.00 Kx total magnification.

XPS was collected on K-ALPHA (Themo Fisher Scientific, USA) with monochromatic X-ray Al K-alpha radiation -10 to 1350 e.v spot size 400 micro at pressure 10-9 mbar with full spectrum pass energy 200 e.v and at narrow spectrum 50 e.v.

To determine the total number of acidic sites (sites.g^{-1}) on the UNP sample calcined at 500°C, temperature-programmed desorption (TPD) of tetrahydrofuran (THF) condensed phase was used. Prior to exposing the probe molecule (THF), 50 mg of the UNP sample was preheated at 350°C for 1 hour in air. About 20 mg of the sample covered with THF was subjected to thermogravimetric analysis (TGA) and differential scanning calorimetry (DSC) analyses at a heating rate of 10°C.min^{-1} in dry N_2 flowing at a rate of 40 ml.min^{-1}, using a 50H Shimadzu thermal analyzer from Japan. The thermal analyzer was equipped with a data acquisition and handling system (TA-50WSI), and α–Al_2O_3 was used as the reference material in DSC measurements.
